# Multiplex SNaPshot for detection of *BRCA1/2 *common mutations in Spanish and Spanish related breast/ovarian cancer families

**DOI:** 10.1186/1471-2350-8-40

**Published:** 2007-06-29

**Authors:** Sandra Filippini, Ana Blanco, Ana Fernández-Marmiesse, Vanesa Álvarez-Iglesias, Clara Ruíz-Ponte, Ángel Carracedo, Ana Vega

**Affiliations:** 1 Unidade de Xenética, Instituto de Medicina Legal & Grupo de Medicina Xenómica, Facultad de Medicina, Santiago de Compostela, Galicia, Spain; 2 Unidad de Medicina Molecular, Fundación Pública Galega de Medicina Xenómica_SERGAS, Hospital Clínico de Santiago de Compostela, Galicia, Spain

## Abstract

**Background:**

It is estimated that 5–10% of all breast cancer are hereditary and attributable to mutations in the highly penetrance susceptibility genes *BRCA1 *and *BRCA2*. The genetic analysis of these genes is complex and expensive essentially because their length. Nevertheless, the presence of recurrent and founder mutations allows a pre-screening for the identification of the most frequent mutations found in each geographical region. In Spain, five mutations in *BRCA1 *and other five in *BRCA2 *account for approximately 50% of the mutations detected in Spanish families.

**Methods:**

We have developed a novel PCR multiplex SNaPshot reaction that targets all ten recurrent and founder mutations identified in *BRCA1 *and *BRCA2 *in Spain to date.

**Results:**

The SNaPshot reaction was performed on samples previously analyzed by direct sequencing and all mutations were concordant. This strategy permits the analysis of approximately 50% of all mutations observed to be responsible for breast/ovarian cancer in Spanish families using a single reaction per patient sample.

**Conclusion:**

The SNaPshot assay developed is sensitive, rapid, with minimum cost per sample and additionally can be automated for high-throughput genotyping. The SNaPshot assay outlined here is not only useful for analysis of Spanish breast/ovarian cancer families, but also e.g. for populations with Spanish ancestry, such as those in Latin America.

## Background

Germline mutations in *BRCA1 *(OMIM 113705) and *BRCA2 *(OMIM 600185) account for familial clustering in the majority of families with both breast and ovarian cancer and in approximately one-half of families with site-specific breast cancer [1,2]. *BRCA1 *and *BRCA2 *are both large genes containing 5,592 and 11,385 nucleotides spread over ~100,000 bases of genomic DNA each. There are more than 1,000 BRCA mutations reported to the Breast Cancer Information Core (BIC) database [3]; many of them are unique but there are also numerous examples of recurrent and founder mutations. Founder mutations have been reported in genetic isolate populations such as Ashkenazi Jewish [4,5], Icelandic [6], Finnish [7], Dutch [8,9] and French Canadian [10], as well as in Slavic countries, including Poland [11].

In Spain, a compilation of BRCA test results from different laboratories shows that five mutations in the *BRCA1 *gene (c.187-188delAG [p.Q23fs], c.330A > G, [p.64-71del], c.5236G > A [p.G1706A], c.5242C > A [p.A1708E], c.589-590delCT [p.S157X]; numbered GeneBank U14680) account for 46.6 % of *BRCA1 *mutations and four in *BRCA2 *(c.3036-3039delACAA [p.K936fs], c.6857-6858delAA [p.E2210fs], c.9254-9258del5 [p.Y3009fs], c.9538-3539delAA [p.K3104fs]; numbered GenBank U43746) account for 56.6 % of the *BRCA2 *mutations [12]. Moreover, an additional founder mutation in the Spanish population, located in *BRCA2 *has recently been observed: c.5374-5377delTATG [p.Y1716fs] (Comunicated by Infante M, et al. at the XXIII Congreso Nacional de Genética Humana, Valladolid, Spain, 2006).

Automated sequencing using standardized procedures represents the "gold standard" system of analysis (complemented by an assay that can detect copy number changes as well). However this procedure is time-consuming and expensive. The identification of fully characterized, recurrent mutations could reduce the time and cost of the analysis. Thus, for instance, Revillion et al. [13] developed a multiplex single-nucleotide primer extension analysis to simultaneous detects 36% of French mutations and 32 % of international ones.

In the present work we describe a rapid, sensitive and low cost assay based on a multiplexed single nucleotide primer extension reaction using SNaPshot dye labeled terminators and capillary electrophoresis to allow rapid detection of approximately 50% of the mutations detected in *BRCA1 *and *BRCA2 *in Spanish families.

## Methods

### Samples and Extraction method

The 48 DNA patient samples included in this study were sent to our laboratory for *BRCA1 *and *BRCA2 *diagnosis. Written informed consent was obtained from each patient. Twenty-four of the 48 DNA samples carried one of the ten recurring germline mutations in the *BRCA1 *and *BRCA2 *genes identified in Spain (*BRCA1*: c.187-188delAG, c.330A > G, c.5236G > A, c.5242C > A, c.589-590delCT; numbered GeneBank U14680; *BRCA2: *c.3036-3039delACAA, c.5374-5377delTATG, c.6857-6858delAA, c.9254-9258del5, c.9538-3539delAA numbered GeneBank U43746). In the remaining 24 samples, no *BRCA1/2 *mutations were present. DNA was extracted from peripheral blood leucocytes by standard procedures.

### SNaPshot multiplex primer design

The primers for PCR amplification and SNaPshot extension reactions (Tables 1 and 2) were both designed to have an annealing temperature around 60°C using Primer3 software [14]. To test for possible repetitive sequences, primers were aligned with the NCBI sequence databases using BLAST [15]. AutoDimer Software [16] was used to test for potential hairpin structures and primer-dimer problems. Primer synthesis was performed by Sigma-Genosys Ltd. (Pampisford, Cambridgeshire, UK). All minisequencing primers were purified by HPLC to remove incomplete primer synthesis products. Co-amplification in the same amplicon was used to detect c.5236 G > C and c.589-590delCT closely positioned in exon 18 of *BRCA1*. The PCR product sizes ranged from 151 bp to 250 bp.

**Table 1:** PCR primer sequences (5'→3')

***Mutation (Predicted effect)***	**PCR primers**	**Size bp**	***Exon***
***BRCA1 *c.187-188delAG (p.Q23fs)**	F ttcgcgttgaagaagtacaaaa R ttccctagtatgtaaggtcaattctg	151	2
***BRCA1 *c.330 A > G (p.64-71*del*)**	F ggctcttaagggcagttgtg R cctactgtggttgcttccaa	235	5
***BRCA1 *c.589-590delCT (p.S157X)**	F ctcttcaggaggaaaagcaca R cctgagacccttacccaattc	186	8
***BRCA1 *c.5236 G > C (p.G1706A)** ***BRCA1 *c.5242 C > A (p.A1708E)**	F ggacagcacttcctgattttg R taaagggaggaggggagaaa	174	18
***BRCA2 *c.3036-3039delACAA (p.K936fs)**	F ttcatgaaacagacttgacttgtg R ttcaaggagatgtccgatttt	208	11
***BRCA2 *c.5374-5377delTATG (p.Y1716fs)**	F tggcttagagaaggaatatttgatg R ctggctcaataccagaatcaag	250	11
***BRCA2 *c.6857-6858delAA (p.E2210fs)**	F tgggaaaagaacaggcttca R gaatgtgtggcatgacttgg	217	11
***BRCA2 *c.9254-9258del5 (p.Y3009fs)**	F tcttccattgcatctttctca R ggtttgtaccggtagttgttga	209	23
***BRCA2 *c.9538-9539delAA (p.K3104fs)**	F gagtttcctttcttgcatcttaaa R gcctgatttggattctggtc	221	25

**Table 2:** Extension primer sequences

***Mutation***	**Extension Primer**	**Size bp**	***Change***
***BRCA1 *c.187-188delAG**	F **gac**tcattaatgctatgcagaaaatcttag	30	**A/T**
***BRCA1 *c.5236 G > C**	F **gact**tgaacggacactgaaatattttctag	30	**G/C**
***BRCA1 *c.330 A > G**	F **gactga**tgtcctttatgtaagaatgatataaccaaa	36	**A/G**
***BRCA2 *c.5374-5377delTATG**	F **gactgactgact**aatactgcagattatgtaggaaattatttg	42	**T/A**
***BRCA1 *c.589-590delCT**	F **gactgactgactgactgactgact**aaaccagtctcagtgtccaactct	48	**C/A**
***BRCA2 *c.3036-3039delACAA**	F **gacgactgactgactgactgactgact**ggttttatatggagacacaggtgataa	54	**A/G**
***BRCA1 *c.5242C > A**	R **acgactgactgactgactgactgactgactgactgact**gctaactacccattttcctccc	60	**T/G**
***BRCA2 *c.9254-9258del5**	F **gacgactgactgactgactgactgactgact**gttaacagaaggaaagagatacagaattt	60	**A/C**
***BRCA2 *c.6857-6858delAA**	F **gacgactgactgactgactgactgactgactgactgact**ctgatgttcctgtgaaaacaaatatag	66	**A/G**
***BRCA2 *c.9538-9539delAA**	F **gagactgactgactgactgactgactgactgactgactgactgact**acgaatgttacaatttactggcaata	72	**A/G**

* Poly(d**GACT**) tail

The extension primer was designed to anneal immediately adjacent to the nucleotide at the mutation site, on either the sense or anti-sense DNA strand, for the detection of *BRCA1 *c.5242C > A mutation we used anti-sense primer. The primer orientation allowed a multiplexed SNaPshot reaction encompassing all the loci to be detected in the absence of intra- and inter-primer complementarities.

### PCR multiplex amplification

The PCR multiplex co-amplified 9 fragments for the 10 *BRCA1/BRCA2 *mutations, with sizes ranging from 174 to 250 bp with a single amplicon encompassing the two exon 18 mutations: c.5242 G > A, and c.589-590delCT of *BRCA1 *gene. Amplification was performed using 5 ng of DNA template, 2 × PCR master mix from QIAGEN Multiplex PCR kit (Qiagen Hilden Germany), 10 × amplification primer mix (2 *μ*M each primer with final concentration of 0.2 *μ*M, optimal for most primer-template systems). Cycling was carried out using a 9700 thermocycler (Applied Biosystems, Foster City, CA, USA). Following a 95°C initial activation step for HotStar Taq DNA polymerase of 15 minutes, a total of 35 cycles were made using the following conditions: 94°C denaturation for 30 seconds, primer annealing at 60°C for 90 seconds, followed by 15 minutes of final extension at 72°C and a 4°C holding step. PCR quality and yield were checked using an Agilent 2100 Expert Bioanalyzer DNA 500/DNA 1000 (Agilent Technologies Inc. Santa Clara, CA USA).

### Minisequencing SNaPshot reaction

After amplification, PCR products required purification to remove primers and un-incorporated dNTPs. This post-PCR purification was performed as follows: 1 *μ*l of PCR product was incubated with 0.5 *μ*l of ExoSapIT (Amershan Pharmacia Biotech) for 15 min at 37°C followed by 15 min at 80°C for enzyme inactivation. The minisequencing reaction was performed using the SNaPshot™ kit in a 9700 thermocycler with 2 *μ*l of SNaPshot ready reaction mix, 0.2 *μ*M of extension primer for each mutation and 1.5 *μ*l of purified PCR products in a 5 *μ*l total volume. Extension used 25 cycles of denaturation at 96°C for 10 seconds, annealing at 50°C for 5 seconds and extension at 60°C for 30 seconds. Detection of extension products is based on four different fluorochromes to assay each base and controlled extension primer sizes (the length of a primer regulated using different sized non-homologous Poly-dGACT tails at the 5' end). Final extension product sizes ranged between 30 and 72 bp (see Table 2). After the minisequencing reaction, a post-extension treatment to remove the 50-phosphoryl group of the ddNTPs helps to prevent unincorporated terminators from comigrating with the extended primers and producing high background signal. For this a 10 *μ*l final volume was treated with 1 *μ*l of SAP (Amersham Biosciences) for 60 min at 37°C, followed by 15 min at 80°C for enzyme inactivation.

### Electroforetic SNaPshot conditions

The minisequencing products (1.5 *μ*l) were mixed with 10 *μ*l of HiDi™ formamide and 0.5 *μ*l of GeneScan-120 LIZ size standard (Applied Biosystems) and denatured at 95°C for 5 minutes. The fluorescently labeled fragments were resolved by capillary electrophoresis on an ABI PRISM 3730 × l Genetic Analyzer (Applied Biosystems). Minisequencing products were injected electrokinetically for 10 seconds at 15 kV and electrophorezed for 20 min at 15 kV and 9 mA at 60°C (default module) in a 36 cm length capillary using POP-7™ polymer with the laser set at a constant power of 9.9 mW. The resulting data was analyzed with GeneMapper™ 3.7 Software (Applied Biosystems).

## Results

**Figure 1:**
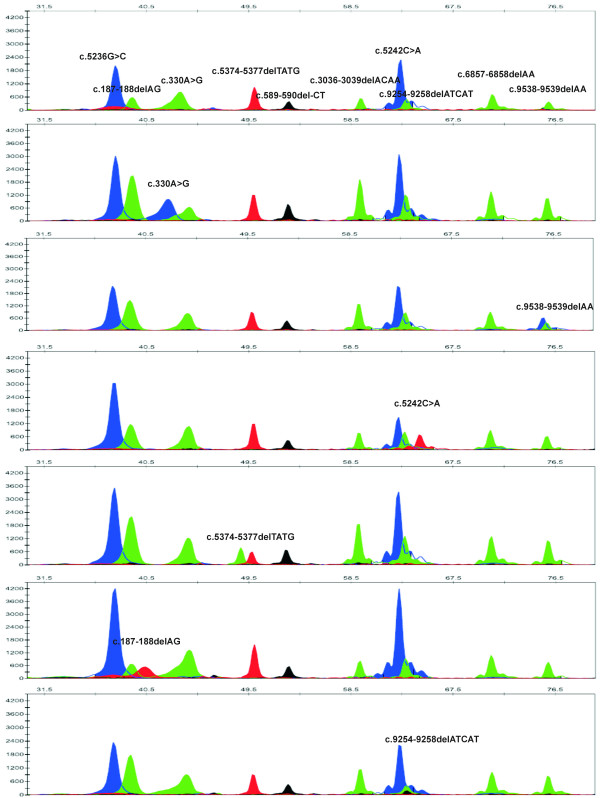
Electropherograms of Genscan Analysis of the SNaPshot reaction. On the top, a control DNA sample with the position and nucleotide of the wild type allele indicated. Following, electropherograms for the most frequents *BRCA1/2 *mutations.

The SNaPshot reaction was performed on samples previously analyzed by direct sequencing and all mutations were concordant. Mutations are usually present in the heterozygous state yielding two peaks, one with the expected size and color and a "mutant" peak with a different color according to the base incorporated (Fig. 1).

The electrophoretic mobility of the extension products depends not only on their lengths, but also on the neighboring nucleotide sequences and on the fluorescent dye used in the reaction.

Therefore, as expected, the detected size determined by the automated sequencer and the real size of certain products were slightly different. It can be tentatively said that to ensure correct differentiation of the extended primer, amplicons should differ by at least six nucleotides in length. Some fragments display stronger fluorescent signal than others in the electropherogram (even after colour compensation with the appropriate matrix) due to variation in minisequencing chemistry. Therefore, mutations are detected by the automated sequencer at different peak heights depending on the terminator and dye label. In general, guanine labeled with dR100 produces a higher signal than the other ddNTPs. However, this fact does not unduly affect the readability of the electropherogram [see also [17]

No false positive results were observed in control samples without mutations, demonstrating the reliability of the procedure and repetitive assays proved the reproducibility of the method.

## Discussion

The genetic analysis of *BRCA1 *and *BRCA2 *is complex and expensive essentially because their length. Nevertheless, the presence of recurrent and founder mutations allows a pre-screening for the identification of the most frequent mutations found in each geographical region. This is the case of the single *BRCA2 *mutation found in the Icelandic population (c.999del5), and the 3 founder Ashkenazi Jews mutations that comprise the genetic test targeted to this population. In contrast to isolated populations the number of mutations that need to be included in the pre-screening process in Spanish population is substantially higher: ten recurrent and founder mutations represent approximately 50 % of the identified familial mutations, and are good candidates to be included in the pre-screening [12, and communication of Infante M, et al. at the XXIII Congreso Nacional de Genética Humana, Spain]. Based on this high-frequency-mutation group, we have developed a SNaPshot reaction to analyze all ten mutations in a single assay. This strategy has numerous advantages. Firstly, a single tube reaction is used for each patient that implies the reduction in term of number of steps and handling. Secondly, high-throughput genotyping is possible as the process can be automated, and the assay is carried out in 96 wells plates. In addition, the interpretation of the peak patterns is very simple and the method is sensitive [see other forensic applications in 18 and 19] and low cost. Lastly, the design is highly flexible: (i) more mutations can be added to the multiplex and (ii) SNaPshot can detect point mutations but also indels, as in our design (some point mutations: c.330A > G or c.5236G > A, and some indels: c.187-188delAG or c.3036-3039delACAA).

The SNaPshot design we propose here represents an advance in the genetic diagnosis of *BRCA1 *and *BRCA2 *Spanish families, since permits the identification of the 46.6 % of *BRCA1 *and more than 56.6 % of the *BRCA2 *families in few hours. Moreover it is not only useful for Spanish patients, but also for those populations with Spanish ancestry such as those from Latin American countries. Many families from Latin America can trace their European ancestry to the period of Spanish colonization, and, in the last century, to migratory movements from diverse regions of Spain. An example of this is the occurrence of two Spanish mutations c.330A > G in *BRCA1 *and c.6857-6858delAA in *BRCA2 *in populations with Spanish ancestry: the former was found in Latin/South American/Caribbean families with Galician ancestors [20,21], and the later mutation was described in one Chilean family with Catalan origins [12].

The utility of our SNaPshot design in Latin American population also applies to recent admixture populations. Thus, for instance, the study of Torres et al. [22] shows that three mutations account for almost 80% of deleterious mutations identified in a Hispanic American cohort from Colombia. Two of these three mutations are frequent in Spanish population and included in our SNaPshot multiplex design we present here, supporting the utility of our design for the initial screening of Latino American breast cancer families. Five of the nine different mutations observed in Chile [23] are in the Top 20 Mutation Frequency of the BIC database [3]. This suggest that a modified SNaPshot considering these Top 20 mutation (some already included in our SNaPshot design) could yield good results for screening in Chile as in other recently admixture populations.

## Conclusion

In summary, here we propose a fast, flexible and cost-effective multiplex assay of the ten most frequent *BRCA1 *and *BRCA2 *mutations in Spain, for the initial screening of Spanish and Spanish ancestry population's breast/ovarian cancer families.

## Competing interests

The author(s) declare that they have no competing interests.

## Authors' contributions

SF performed the genetic design, carried out the genetic analysis and drafted the manuscript. AB and AFM helped with the genetic analysis of the samples and drafted the manuscript. VAI help in the methodological design. CRP and AC helped to draft and revised the manuscript. AV conceived the study, participated in its design and coordination, and wrote the manuscript. All authors read and approved the final manuscript.

## Pre-publication history

The pre-publication history for this paper can be accessed here:


